# Complete Genome Sequence of *Bradyrhizobium* sp. ORS285, a Photosynthetic Strain Able To Establish Nod Factor-Dependent or Nod Factor-Independent Symbiosis with *Aeschynomene* Legumes

**DOI:** 10.1128/genomeA.00421-17

**Published:** 2017-07-27

**Authors:** Djamel Gully, Albin Teulet, Nicolas Busset, Nico Nouwen, Joël Fardoux, Zoe Rouy, David Vallenet, Stéphane Cruveiller, Eric Giraud

**Affiliations:** a IRD, Laboratoire des Symbioses Tropicales et Méditerranéennes, UMR IRD/SupAgro/INRA/UM/CIRAD, Campus International de Baillarguet, TA A-82/J, Montpellier, France; b Commissariat à l’Energie Atomique, Direction des Sciences du Vivant, Institut de Génomique, Génoscope, Évry, France

## Abstract

Here, we report the complete genome sequence of *Bradyrhizobium* sp. strain ORS285, which is able to nodulate *Aeschynomene* legumes using two distinct strategies that differ in the requirement of Nod factors. The genome sequence information of this strain will help understanding of the different mechanisms of interaction of rhizobia with legumes.

## GENOME ANNOUNCEMENT

The photosynthetic *Bradyrhizobium* sp. strain ORS285 is able to induce the formation of nitrogen-fixing nodules on the root and stem of some aquatic tropical legumes of the *Aeschynomene* genus (1). In contrast to the previously sequenced photosynthetic *Bradyrhizobium* strains (BTAi1 and ORS278) (2), this strain does contain the canonical *nodABC* genes required for the synthesis of Nod factors (NF) and displays a broader host range (3). Depending on the host plant, two symbiotic processes can be distinguished: a classical one involving NF perception, and an alternative one which is NF independent and for which the symbiotic signal molecules remain to be disclosed (2, 4). *Bradyrhizobium* sp. ORS285 is therefore an interesting model strain in comparative studies of these two distinct symbiotic mechanisms (4).

A draft genome sequence of the ORS285 strain has been previously obtained using 454-pyrosequencing technology, but the presence of a high number of transposase genes and repetitive DNA sequences resulted in a fragmented genome (301 contigs) which made analysis of the regions containing symbiotic genes difficult (5).

In this study, we have obtained the complete genome sequence of *Bradyrhizobium* sp. strain ORS285 using the Pacific BioSciences (PacBio) sequencing technology. Genomic DNA libraries were prepared using the Pacific Biosciences 20-kb library preparation protocol. A total of 60,640 polymerase reads with a mean read length of 14,753 bp were generated, which led to a total of 894.59 Mb, with an average coverage of 114-fold. *De novo* assembly of the read sequences was performed using continuous long reads according to the Hierarchical Genome Assembly Process (HGAP) version 3 workflow (DevNet; Pacific Biosciences) as available in the SMRT Analysis software version 2.3.0. The circularization of contigs was performed with Minimus2 software (Amos package). The sequence was polished sequentially with RS_Resequencing.1 software (SMRT Analysis version version 2.3.0) and Pilon software (version 1.21) using available transcriptomic data (HiSeq 2000; Illumina).

The genome of *Bradyrhizobium* sp. ORS285 comprises one circular chromosome of 7,797,098 nucleotides, with an average G+C content of 65.19%. Annotation of the *Bradyrhizobium* sp. ORS285 genome was performed using the MicroScope platform (6) and subsequently verified manually. A total of 6,983 coding sequences (CDSs) and 51 tRNAs were predicted. In contrast to the genomes of ORS278 and BTAi1, a clear symbiotic island of 262 kb was identified. To our knowledge, this is the smallest symbiotic island described for a *Bradyrhizobium* strain. It contains *nod* genes, a type III secretion system (T3SS) gene cluster, genes involved in tricarboxylic acid transport, and numerous transposase elements. Remarkably, the *nif* genes are not located in the symbiotic island but are located in a different region of the genome. Comparing the complete genome sequence of *Bradyrhizobium* sp. ORS285 with other bradyrhizobial genomes will facilitate the identification of the symbiotic genes governing the NF-dependent and NF-independent processes.

### Accession number(s).

This genome sequence has been deposited in ENA/GenBank/DDBJ under the accession no. LT859959.
